# The prediction of single-molecule magnet properties via deep learning

**DOI:** 10.1107/S2052252524000770

**Published:** 2024-02-01

**Authors:** Yuji Takiguchi, Daisuke Nakane, Takashiro Akitsu

**Affiliations:** a Department of Chemistry, Tokyo University of Science, 1-3 Kagurazaka, Shinjuku-ku, Tokyo 1628601, Japan; Sun Yat-Sen University, China

**Keywords:** single-molecule magnets, deep learning, Cambridge Structural Database, salen-type complexes

## Abstract

This paper uses deep learning to present a proof-of-concept for data-driven chemistry in single-molecule magnets (SMMs). Previous discussions within SMM research have proposed links between molecular structures (crystal structures) and single-molecule magnetic properties; however, these have only interpreted the results. Therefore, this study introduces a data-driven approach to predict the properties of SMM structures using deep learning. The deep-learning model learns the structural features of the SMM molecules by extracting the single-molecule magnetic properties from the 3D coordinates presented in this paper. The model accurately determined whether a molecule was a single-molecule magnet, with an accuracy rate of approximately 70% in predicting the SMM properties. The deep-learning model found SMMs from 20 000 metal complexes extracted from the Cambridge Structural Database. Using deep-learning models for predicting SMM properties and guiding the design of novel molecules is promising.

## Abstract 2

This work involves extraction of salen metal complexes from the Cambridge Structural Database for deep learning to examine the 3D structural features that allow such complexes to act as single-molecule magnets. This research attempts to link a crystal structure database as big data with the molecular design of nanomaterials using artificial intelligence. The approach pioneers the future secondary use of similar crystal structure data.

## 1. Introduction

Single-molecule magnets (SMMs) are metal complexes that exhibit magnetic relaxation behaviour like bulk ferromagnets as single molecules (Gatteschi *et al.*, 2006 ▸; Winpenny & Aromí, 2006 ▸). Molecular-sized nanoscale magnets are expected to find applications in high-density memory and quantum-computing devices (Leuenberger & Loss, 2001 ▸; Eliseeva & Bünzli, 2011 ▸). The effective activation energy barrier (*U*_eff_) is typically used to assess magnetization reversal in SMMs (Woodruff *et al.*, 2013 ▸). Owing to the relationship shown in equation (1) between *U*_eff_, zero-field splitting parameter *D* and total spin quantum number *S* of the molecule (Zhang *et al.*, 2013 ▸), *D* and *S* are essential keywords in SMM molecule designs:

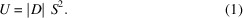

Although early strategies concentrated on multinuclear complexes of transition metals, such as those represented by the Mn_12_ cluster (Sessoli *et al.*, 1993 ▸), mononuclear lanthanide complexes now play a central role (Zhang *et al.*, 2013 ▸; Dey *et al.*, 2018 ▸). *Ab initio* calculations have demonstrated that highly axial [DyO]^+^ ions in lanthanide metal complexes display *U*_eff_ values above 3000 K (Ungur & Chibotaru, 2011 ▸). Molecules with *U*_eff_ values of approximately 2000 K have been reported (Randall McClain *et al.*, 2018 ▸). However, it is exceedingly challenging to fabricate molecules that exhibit high *U*_eff_ values because the *U*_eff_ of traditional SMMs is typically in the range of tens to hundreds of kelvin (Tang & Zhang, 2015 ▸; Liu *et al.*, 2015 ▸; Parmar *et al.*, 2021 ▸). *U*_eff_ is affected by quantum tunnelling and antiferromagnetic interactions, and achieving control over the structure of lanthanide metal complexes in crystals is challenging. Currently, there are no established guidelines for SMM design.

In the theoretical field, discussions using magnetic properties obtained from the complete active space self-consistent field (CASSCF) method (Roos & Malmqvist, 2004 ▸) using the *Molcas* program (Aquilante *et al.*, 2010 ▸) provide insight into the origin of SMMs. In addition, some techniques leverage the CASSCF method to estimate *U*_eff_ directly (Aravena, 2018 ▸; Yin & Li, 2020 ▸). Nevertheless, these methods require substantial computational resources and time, and selecting computational conditions is a significant bottleneck. These algorithms are unsuitable for extensive calculations that require a vast search space, such as molecular design, because they have only been utilized to interpret experimental outcomes.

Recently, interest in data-driven chemistry and theory-predicate research, including machine learning, has grown (Engel, 2006 ▸; Mitchell, 2014 ▸). The *Chemical Reviews* journal released a special issue on ‘Machine Learning at the Atomic Scale’ (Ceriotti *et al.*, 2021 ▸). The Tsuda group at RIKEN provided instances of newly designed fluorescent materials successfully developed using AI and density functional theory (Sumita *et al.*, 2018 ▸; Sumita *et al.*, 2022 ▸). However, data-driven research at the laboratory level has remained unsuccessful. Machine learning of metal complexes has predominantly focused on investigating hexacoordinated and tetracoordinated complexes (Nandy *et al.*, 2021 ▸). However, addressing the structural changes caused by the spin multiplicity and similarity definitions is crucial for effectively performing machine learning on these complexes. Thus, extending conventional organic molecule machine learning to all elements is insufficient (Janet *et al.*, 2019 ▸). It is unclear what AI can do in the SMM field, in which lanthanide ions and metal complexes with complex structures play central roles.

Here, we concentrate on the SMM research and present a proof-of-concept for the data-driven chemistry of lanthanide metal complexes and metal complexes with intricate structures. A dataset was compiled from papers on salen-type SMMs from the preceding decade, and deep learning was used to predict SMM behaviour. The SMM characteristics were predicted using approximately 20 000 crystal structures of the Schiff base metal complexes extracted from the Cambridge Structural Database (CSD). Although many statistical investigations (Duan *et al.*, 2022 ▸) and systematic reviews (Zhang *et al.*, 2013 ▸; Dey *et al.*, 2018 ▸) of SMMs have been conducted, this is the first report of deep learning on the molecular structures of SMMs. From the CSD, numerous molecules predicted to display SMM behaviour did indeed exhibit SMM behaviour, thereby demonstrating the capacity of the model to understand SMM features and ‘discover’ SMMs. Such indications imply that implementing AI in SMM research could introduce a novel paradigm shift akin to AlphaGo’s AI (Silver *et al.*, 2016 ▸) that surpassed human players.

## 2. Methods

### 2.1. Datasets

Molecules reported as SMMs comprise numerous chemical species like phthalocyanine complexes, metallocenes and polyoxometalate complexes (Duan *et al.*, 2022 ▸). Salen-type metal complexes were selected as the focus of this study. Salen-type ligands can be effortlessly synthesized from aldehydes and amines to form complexes with various 3*d* and 4*f* metals. Several SMM structures have been reported, including mononuclear, binuclear and 3*d*–4*f* multinuclear complexes (Liu *et al.*, 2015 ▸). From these varied structures, it is expected that structures specific to SMMs can be identified.

The dataset was created using approximately 800 papers from 2011–2021 that were found using the keywords ‘salen + SMM’ using Google Scholar. Data on the crystal structures and whether they exhibited SMM behaviour were collected from these studies, and a dataset was created. Non-SMM molecules were defined as those that did not exhibit magnetic relaxation behaviour according to AC magnetic susceptibility measurements. Tunnelling effects are suppressed under an appropriate DC magnetic field, increasing the likelihood of SMM behaviour. If the measurement results exist under both zero and DC magnetic fields, priority is given to the results under the zero magnetic field.

CIF files were obtained from the CSD (Groom *et al.*, 2016 ▸). The crystal structures were verified using the *Mercury* crystal drawing software (Macrae *et al.*, 2020 ▸). The molecular structures were then converted to molecular structure files (XYZ files) by excluding non-pertinent molecules such as crystal water and were utilized as coordinate data. The explanatory variables were the molecular structures retrieved from the crystal structures, while the objective variable was the SMM behaviour.

### 2.2. Input representation

The input representations of the molecular structures typically include molecular descriptors, graphs and SMILES (Elton *et al.*, 2019 ▸). This study utilized 3D images (voxels) as input representations to represent the metal complexes. Although their memory-intensive requirements compared with other input representations, voxel usage is expected to retain the 3D information of the molecules. Their use has been demonstrated for compounds of different sizes and structures, including organic molecules, inorganic solids and proteins (Ryan *et al.*, 2018 ▸; Amidi *et al.*, 2018 ▸; Kuzminykh *et al.*, 2018 ▸; Li *et al.*, 2021 ▸; Park & Seok, 2022 ▸). These results represent the structural features of metal complexes with different central metals and structures.

**Figure 1:**
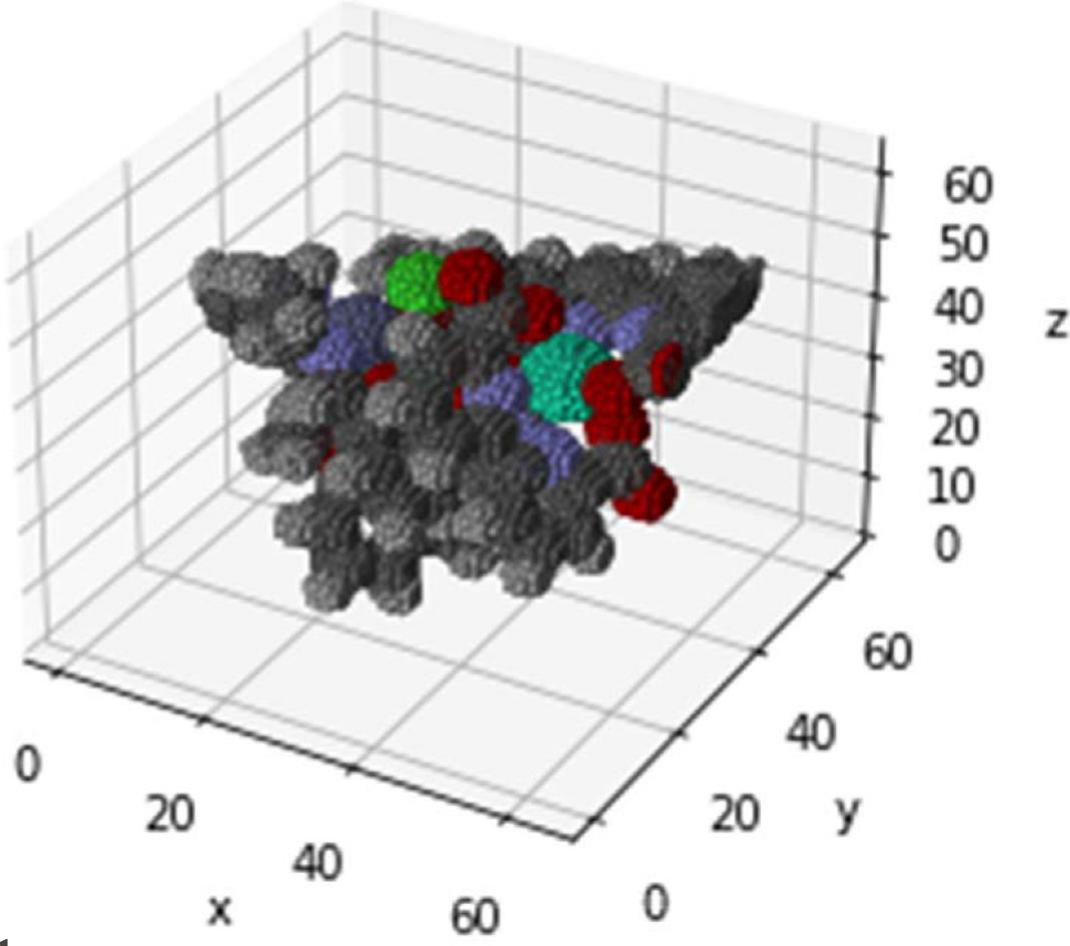
Voxel diagram of the metal complex. Create a 64 × 64 × 64 × 3 array and specify the colour of each element [R, G, B] for the voxels within a radius *r* centred on the atom position. [0,0,0] is specified for empty space. In addition, the size of the sphere was changed for each element in proportion to the ionic radius. It is an intuitive and highly versatile descriptor, requiring no special pre-processing.

The dimensions of the voxels were set to 64 × 64 × 64, with the element type represented by the assigned colour (RGB value) for each element. One side of the voxel was established at 12 Å to preserve the molecular structure. 3D molecular images were created by placing spherical voxels at 3D atomic coordinates obtained from the XYZ file, and 3D molecular images were created (Fig. 1 ▸). A balance between voxel resolution and computational resources must be achieved, and an appropriate voxel size must be determined via trial-and-error experimentation.

### 2.3. Model architecture

**Figure 2:**
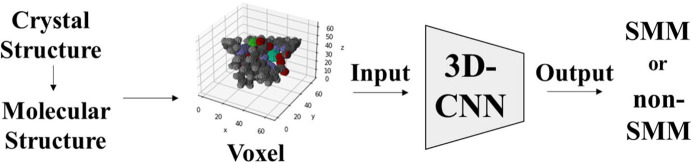
Conceptual diagram of SMM prediction. The CIF file was edited using crystal drawing software such as *Mercury*. Crystal water contained in the crystal was removed, and the structure of only the target complex molecule was converted into an XYZ file. The XYZ file is converted into voxels, and 3D-CNN predicts whether it is SMM or non-SMM.

A 3D convolutional neural network (3D-CNN) was used as the deep-learning model to create a binary classification model that predicts whether a molecule is an SMM based on its molecular structure (Fig. 2 ▸); this multilayer neural network consists of convolutional layers and fully connected layers. The input image is output as a scalar value between 0 and 1 by the last fully connected layer after the 3D convolutional layer extracts the 3D image features (Jogin *et al.*, 2018 ▸). In this study, SMMs were set to 0 and non-SMMs to 1; however, which was 0 did not affect the results. ResNet with residue connections (He *et al.*, 2019 ▸) was selected as the network structure owing to its impressive image classification accuracy. ResNet can effectively construct deep CNN models with many layers through residual connections between the convolutional layers. The model comprises multiple Res-blocks connected via the GAP layer to fully connected layers. The Res-blocks employ the activation function pre-positioning type, with the ReLU layer positioned before the confluence. Furthermore, a bottleneck-type structure was adopted for these blocks to reduce computational demands. Batch normalization (Bjorck *et al.*, 2018 ▸) and a 20% dropout (Gal & Ghahramani, 2015 ▸) were used to prevent overlearning. The ReLU function (Agarap, 2019 ▸) was used as the activation function, and the sigmoid function (Cybenko, 1989 ▸) was used as the final layer.

### 2.4. Model training

As the dataset used in this study was unbalanced data with SMM:non-SMM of 2:1, undersampling (Mohammed *et al.*, 2020 ▸) was used to reduce the SMM data to eliminate data unbalances. The dataset was split 6:2:2 into training, validation and test data. The CNN was trained with the Adam optimiser using AMSGrad (Reddi *et al.*, 2019 ▸) with hyperparameters ɛ = 1 × 10^−7^, β_1_ = 0.9 and β_2_ = 0.999. The learning rate was reduced from an initial value of 1 × 10^−2^ to 1 × 10^−5^ using the cosine reduction rate (Loshchilov & Hutter, 2016 ▸). The batch size was set to eight to account for GPU memory. The loss function used was the cross-entropy error (Bishop, 1995 ▸), which was trained to minimize. The correct response rate and AUC were used as model evaluation indices. Data augmentation (Shorten & Khoshgoftaar, 2019 ▸) was performed during training to reduce over-learning. As the molecular structure was obtained in atomic coordinates, voxels were generated after rotation, scaling and translation to 3D coordinates. By performing data augmentation, the model is expected to prevent overlearning and acquire rotational and translational invariance. The CNN was trained for 1000 epochs. These hyperparameters were derived by trial and error.

### 2.5. Software and libraries

All the programs were implemented using Python 3. *TensorFlow* 2.0 (Abadi *et al.*, 2016 ▸) was used as the machine learning library. The *NumPy* (Harris *et al.*, 2020 ▸) and *SciPy* (Virtanen *et al.*, 2020 ▸) libraries were used for coordinate transformation and voxel generation. Open Babel (O’Boyle *et al.*, 2011 ▸) was used to transform the molecular structure files, and CSD Python API (Moghadam *et al.*, 2020 ▸) was used to obtain and manipulate the crystal structures. The model training was performed on a Windows 10 OS workstation with an Intel Xeon E5-2620 v3 (12 cores, 24 threads) CPU, 128 GB RAM and a GPU (NVIDIA Tesla K40, 12GB).

## 3. Results and discussion

### 3.1. Dataset overview

**Figure 3:**
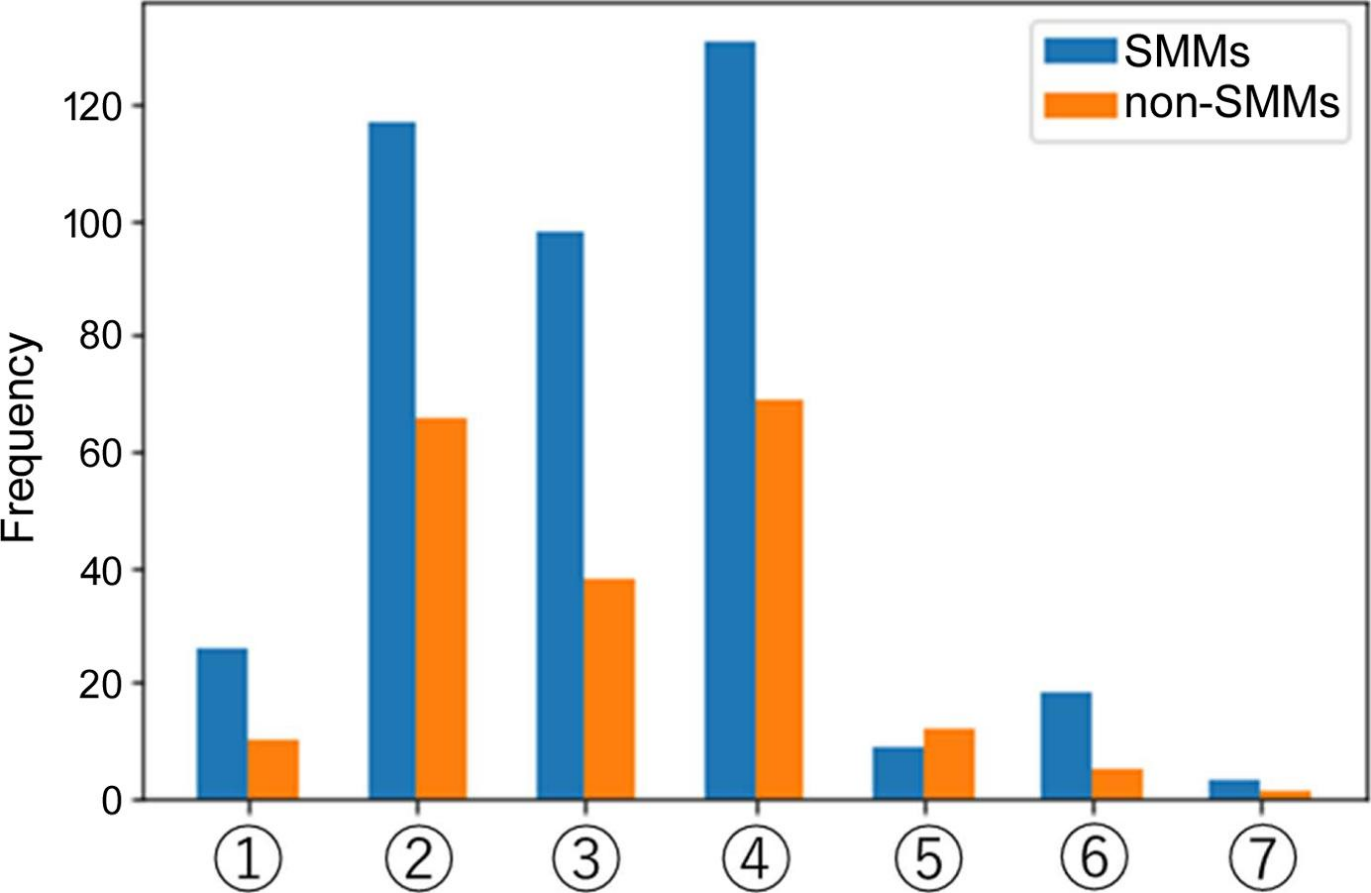
Histogram of SMM and non-SMM molecules included in the dataset. Blue indicates SMM molecules, and orange indicates non-SMM molecules. (1) Complexes consisting of a single lanthanoid ion. (2) Homonuclear lanthanide ion dinuclear complexes. (3) Homonuclear transition metal dinuclear complexes. (4) 3*d*–4*f* complexes. (5) 4*d*–4*f* complexes or 5*d*–4*f* complexes. (6) Complexes consisting of a single transition metal. (7) Complexes containing actinide ions (uranium).

A histogram of the metal complexes is presented in Fig. 3 ▸ to determine the composition of the molecules included in the dataset. This classification is based on seven categories: (i) complexes consisting of a single lanthanide ion, (ii) homonuclear lanthanide ion binuclear complexes, (iii) homonuclear transition metal binuclear complexes, (iv) 3*d*–4*f* complexes, (v) 4*d*–4*f* or 5*d*–4*f* complexes, (vi) complexes consisting of a single transition metal, and (vii) actinide ions (uranium). Histograms illustrating the number of atoms and nuclei in the SMMs and non-SMMs are provided in the supporting information (Figs. S1 and S2).

Figs. 3 ▸, S1 and 2 indicate that the differences were not pronounced depending on the number of atoms or nuclei. Histograms depicting complexes containing lanthanide ions show that those containing Dy ions display a significant proportion of SMM properties (Fig. S2a), consistent with several previous results (Zhang *et al.*, 2013 ▸; Dey *et al.*, 2018 ▸). Complexes containing Dy, Gd and Tb ions constituted a significant proportion of the data. However, for the other lanthanide ions, the quantity of data was minimal, and no distinctions in the SMM properties due to elemental variations were discernible. For the 3*d*–4*f* complexes, Dy–Zn complexes are the most prevalent molecules with SMM properties, and Dy–Cu, Dy–Ni and Tb–Zn complexes are also included. Alternatively, Dy–*d* complexes are still prevalent in molecules lacking SMM properties. However, note that Tb–Cu complexes were the most common (Figs. S3 and S4). It is challenging to use traditional machine learning to forecast SMM properties because of the difficulty in identifying differences by element or number of nuclei and the presence of Dy complexes that do not exhibit SMM properties.

### 3.2. Learning results

**Figure 4:**
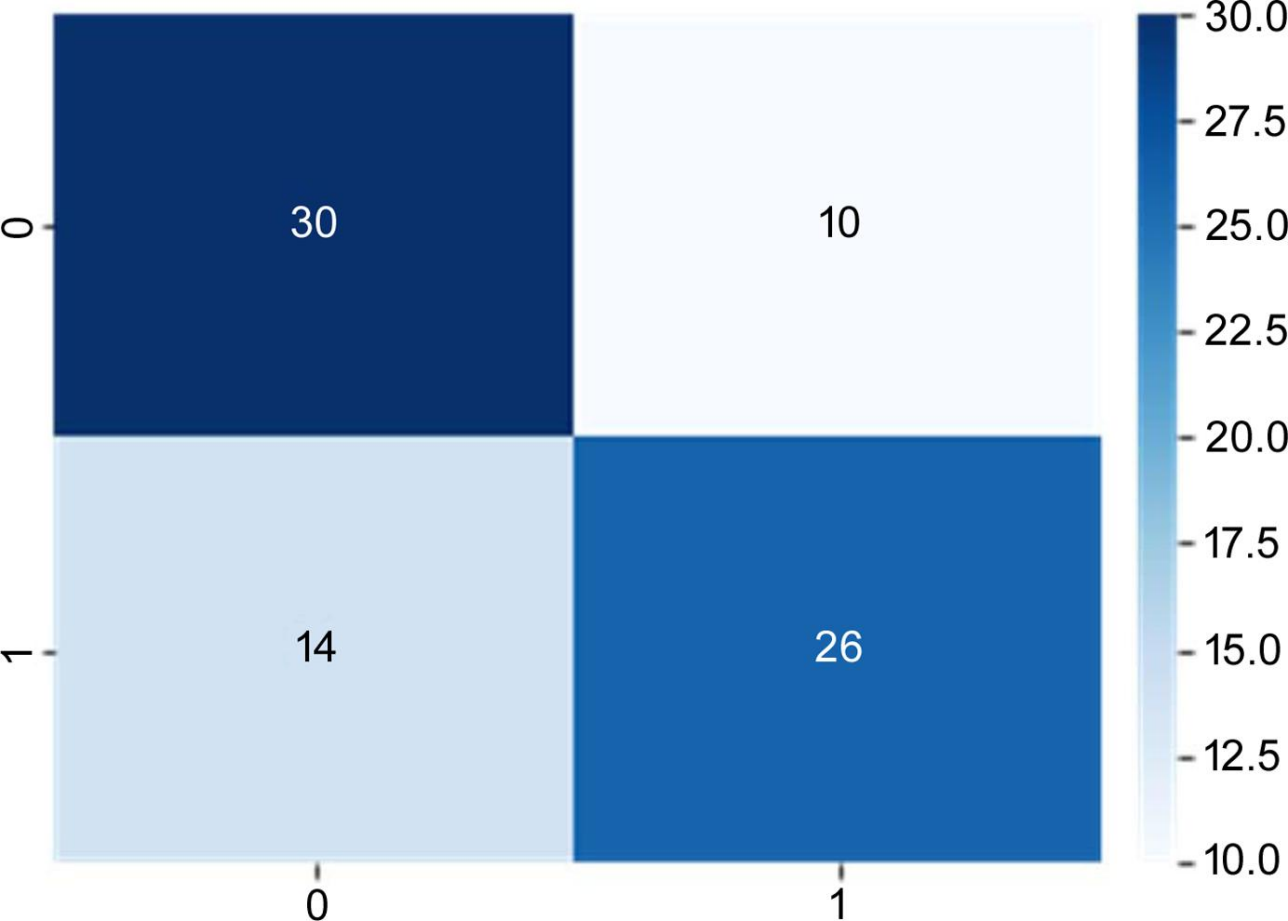
Confusion matrix in test data.

The learning curve is illustrated in Fig. S5, indicating positive progress as the loss decreases with learning. The similarity between the training and generalization errors suggests that the deep-learning model is robust to unknown data. The final accuracy and AUC were approximately 70% (Table 1 ▸). The confusion matrix illustrated in Fig. 4 ▸ exhibits substantial values for the diagonal components. Distinguishing SMMs from molecular structures alone is challenging, even for the human eye. Notably, the deep-learning model demonstrated 70% accuracy, and the prediction in this study was based solely on voxel information generated from the type of element and its positional relationship.

It is expected that enhancing the descriptors and refining the learning model will result in improved accuracy. In particular, utilizing spin and orbital angular momentum is crucial in magnetism. Previous studies have employed charge distributions and spin densities to obtain voxel information (Kuzminykh *et al.*, 2018 ▸; Ghosh *et al.*, 2019 ▸; Casey *et al.*, 2020 ▸). Quantum chemical calculations can determine these values. However, such calculations require significant computational time and resources when dealing with chemical species containing transition and lanthanide metals. These calculations are unfeasible for studies involving hundreds or tens of thousands of molecules, like in this study. In addition, it would be desirable to compare the performance with the results obtained using graphs, molecular descriptors and voxels. However, numerous methods are inadequate for molecules containing metal ions, necessitating the renovation of programs. Unfortunately, this action was not performed in the current investigation because of time restrictions. Further studies are required to address this issue.

### 3.3. Visualization of learning results

**Figure 5:**
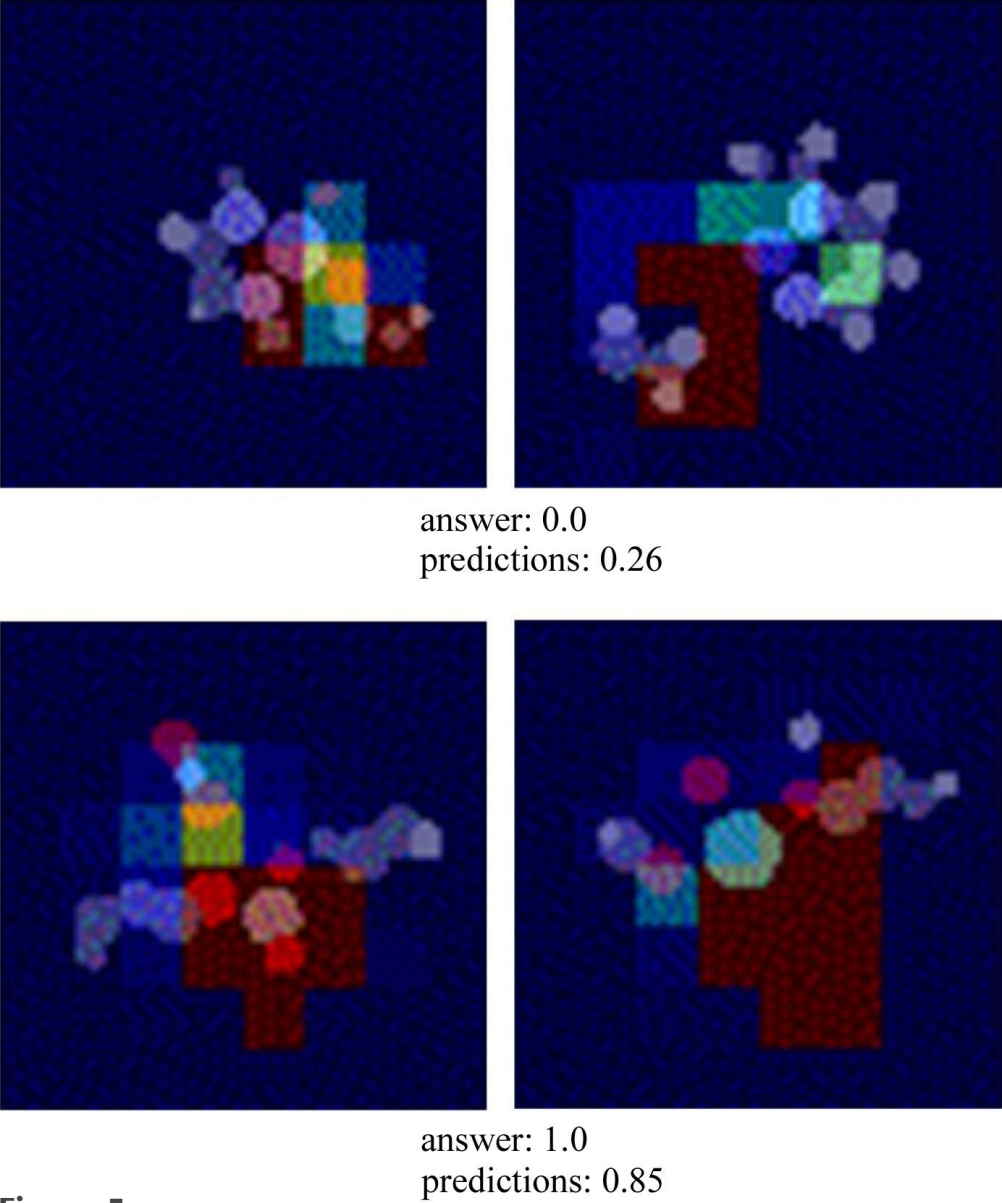
Visualization of CNN prediction results using Grad-CAM. The red blocks in the image are the parts that make a significant contribution when CNN makes predictions. It is obtained as voxels 64 × 64 × 64 in size, but was divided into 64 2D images of 64 × 64 for plotting.

Grad-CAM (Selvaraju *et al.*, 2017 ▸) was used to visualize the area of focus of the model for the inputs. The Grad-CAM diagram indicates that the model is concentrated near the central metal (Fig. 5 ▸). Notably, the model recognizes that the central metal initiates magnetism in metal complexes. Fig. S6 demonstrates the successful and unsuccessful predictions, and the model does not focus on significantly misguided areas, even in failed predictions. Furthermore, this model is not expected to focus on particular ligands or functional groups to perform optimally in unfamiliar molecular structures.

In contrast, we expected to discover new structural features that would go beyond the thinking of chemists; however, for this study, we could only obtain the same insights. The opaque nature of deep-learning predictions impedes thorough analysis, leading to tentative conclusions. However, commonly employed chemoinformatics techniques [graph-neural networks (GNNs) and recurrent neural networks (RNNs)] frequently lack visualization methods compared with CNNs. This attribute of CNNs is advantageous for property prediction, rendering it a potent technique in fields lacking a comprehensive understanding of the reaction mechanism, for example in protein–ligand docking and catalysis studies.

### 3.4. Proof of SMM search with the CSD

Magnetic measurements were conducted in the solid state; therefore, the SMM molecular design must account for the molecular structure within the crystal structure. Predicting the crystal structure of the metal complexes is necessary to design SMM molecules from scratch. However, a method to achieve this goal is yet to be established. Fortunately, the CSD contains one million crystal structures, of which only a small proportion have undergone AC susceptibility measurements. Predicting the SMM behaviour of the crystal structures registered in the CSD was validated as a model case for designing SMM molecules using deep learning.

**Figure 6:**
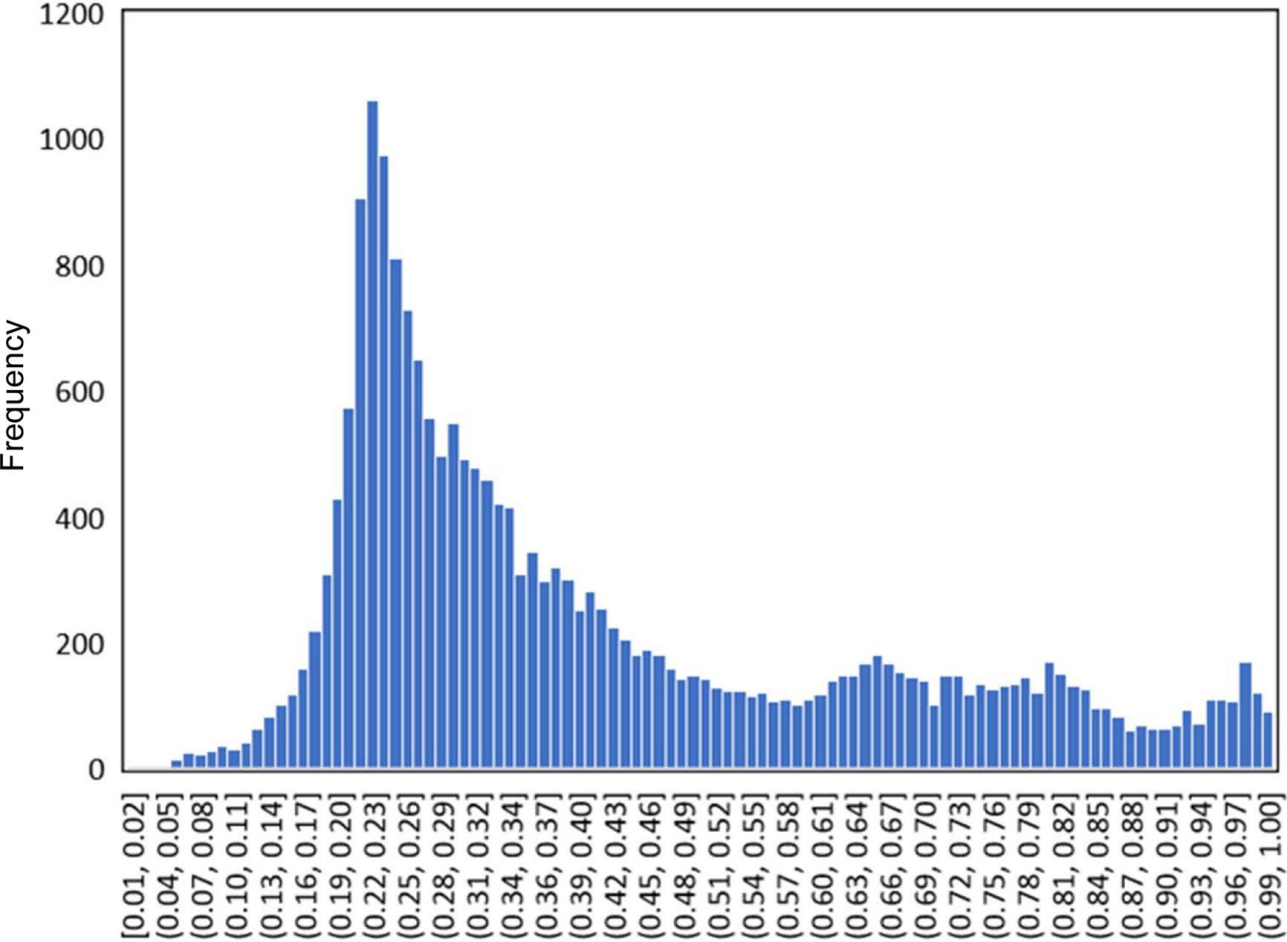
Histogram of predicted SMM properties for approximately 20 000 molecules obtained from the CSD. The closer the frequency is to 0, the more SMM-like it is predicted to be.

Approximately 20 000 crystal structures of metal complexes containing Schiff bases were obtained using the program *ConQuest*. Using the CSD Python API, the molecular structures of the metal complexes were extracted from the crystal structures by removing crystalline water and other components. The SMM behaviours of the molecular structures predicted by the learned model are shown in Fig. 6 ▸. The focus was on the top 10 molecules predicted to be the most SMM-like and the bottom 10 predicted to be the least SMM-like. The original publications were searched using CCDC numbers, and the pivotal metal and magnetic measurements are summarized in Tables 2 ▸ and 3 ▸. Numerous molecules predicted to be SMMs were confirmed to exhibit SMM behaviour. Conversely, many molecules predicted to be non-SMMs have not been measured for AC susceptibility.

Many of the predicted SMM molecules had structures comprising multinuclear Dy complexes. According to equation (1), a larger total number of spins in the complex leads to a more significant value of *U*_eff_. This approach is similar to earlier approaches that enhance the spin multiplicity *S* using multinuclear complexes of *d*-metals. Previous studies have shown that symmetry reduction occurs with increased nuclei, and the highest *U*_eff_ value was 40 K for ZEZYIO (Lin *et al.*, 2012 ▸). This study conducts learning based on the presence or absence of SMM behaviour, with no consideration given to the height of *U*_eff_ for simplicity. However, to identify the more promising SMMs, a dataset that considers *U*_eff_ should be able to select the best SMMs.

It is important to emphasize that the molecular structure data for the 20 000 molecules used in this study did not include the data used in training. Moreover, while salen-type ligands were prominent in the training phase, a more comprehensive range of ligands (Schiff bases) was included in the 20 000 data points. Thus, the deep-learning model solely predicted unknown molecules without knowing the answers, ultimately selecting the SMM molecules from the CSD. For instance, novel SMM molecules may be uncovered by re-synthesizing molecules that have been predicted to be SMMs but have not been subjected to AC susceptibility and magnetic measurements. The deep-learning-based SMM research model established in this study serves as a practical data-driven chemistry framework.

As referenced in previous research (Sumita *et al.*, 2018 ▸, 2022 ▸), for AI-assisted molecular design, it is recommended to use data-independent approaches that involve both (1) AI-generated molecular structures and (2) prediction of properties via quantum chemical calculations. AI generates extensive candidate molecules, ranging from tens to hundreds of millions, and property predictions are performed via large-scale supercomputing. The latter is facilitated using supercomputers to predict computational properties. Electronic structure calculations of organic molecules in solution are highly accurate and have successfully controlled UV absorption and created fluorescent molecules (Sumita *et al.*, 2018 ▸, 2022 ▸). However, solid-state studies on the magnetism of metal complexes encounter two challenges. The first is the obstacle in predicting the crystal structures of metal complexes (Desiraju, 2013 ▸; McDonagh *et al.*, 2019 ▸). The second is the challenge of simulating magnetism. Novel breakthroughs must be made to resolve these issues. As a compromise, this study designs SMM molecules using molecular structures from a crystal database and data-driven prediction by deep learning. However, the data are limited, and the accuracy of the results depends on the quantity of available data. Moreover, because the results were data-dependent, they were not significantly superior to those obtained by conventional molecular design by conventional chemists. Furthermore, it is not feasible to acquire innovative SMMs solely by predicting molecules with well defined crystal structures. Further research is required in the future.

## 4. Conclusions

This study conducted deep learning using experimental data from SMM studies and molecular structures derived from crystal structures. The deep-learning model successfully acquired knowledge of the structural features of the SMM and accurately predicted the SMM behaviour using only 3D structure and magnetic data. We have demonstrated that deep-learning models can identify SMM molecules within the CSD. This study presents a data-driven approach to experimentation instead of relying on subjective evaluations based on intuition and experience. With further developments and the integration of generative AI and first-principles calculations, unprecedented SMMs can be achieved using deep-learning methods.

## Supplementary Material

Supporting table and figures. DOI: 10.1107/S2052252524000770/yc5046sup1.pdf

## Figures and Tables

**Table 1:** Correct answer rate and AUC on training, validation and test data

	ACC	AUC
Train	0.7250	0.8185
Valid	0.6875	0.7758
Test	0.7000	0.7269

**Table 2:** The top 10 molecules predicted most likely to be SMM ‘No’ if reported not, and ‘–’ if AC susceptibility measurements were not performed.

	CCDC	Score	SMM	Metal	Reference
1	QURMUN	0.0076	Yes	Dy18Zn12	Stavgianoudaki *et al.* (2016 ▸)
2	VUVKEE	0.0189	No†	Dy10	Das *et al.* (2015 ▸)
3	REYYEB	0.0228	Yes	Dy5Mn4	Alexandropoulos *et al.* (2013 ▸)
4	RECPIB	0.0289	Yes	Dy6	Lin *et al.* (2017 ▸)
5	ZEZYIO	0.0327	Yes	Dy6	Lin *et al.* (2012 ▸)
6	LOSVOG	0.0331	Yes	Dy9	Zou *et al.* (2015 ▸)
7	OPEFOH	0.0441	No	Dy6	Schlittenhardt *et al.* (2021 ▸)
8	EZEZUG	0.0453	No†	Dy12	Li *et al.* (2016 ▸)
9	YALRAF	0.0472	–	UCs	Cametti *et al.* (2005 ▸)
10	OPEGAU	0.0475	No	Dy6	Schlittenhardt *et al.* (2021 ▸)

† Material exhibits magnetic relaxation behaviour under a DC magnetic field.

**Table 3:** The bottom 10 molecules predicted least likely to be SMM ‘No’ if reported not, and ‘–’ if AC susceptibility measurements were not performed.

	CCDC	Score	SMM	Metal	Reference
1	PIVGAD	0.9990	–	Nd2Cu2	Gheorghe *et al.* (2007 ▸)
2	TOKRAO	0.9990	No	Ni15	Muche *et al.* (2014 ▸)
3	VICQUV	0.9991	–	Nd2	Zou *et al.* (2013 ▸)
4	VAKLEY	0.9994	–	Nd	Ling *et al.* (1989 ▸)
5	YUTCAS	0.9994	–	Nd2Zn2	Lü *et al.* (2010 ▸)
6	YEBPOL	0.9994	–	PrCu2	Gheorghe *et al.* (2006 ▸)
7	XUXNEK	0.9995	–	Pr2Cu2	Pointillart *et al.* (2010 ▸)
8	SIGZIU	0.9995	–	Nd3Zn6	Song *et al.* (2018 ▸)
9	SIGZEQ	0.9996	–	Nd8Zn12	Song *et al.* (2018 ▸)
10	TIYGUF	0.9998	–	Nd6Cd18	Yang *et al.* (2013 ▸)
