# Supplementary material for: The prediction of single-molecule magnet properties via deep learning
Source: IUCrJ. 2024 Feb 1;11(Pt 2):182–9. doi: 10.1107/S2052252524000770 (PMC10916298; doi:10.1107/S2052252524000770)
Supplement: Supplementary file 1 [file m-11-00182-sup1.pdf]

IUCrJ

Volume 11 (2024)

Supporting information for article:

The prediction of single-molecule magnet properties via deep learning

Yuji Takiguchi, Daisuke Nakane and Takashiro Akitsu

Table S1

Table S2 **Table S1** Detailed information for each layer.

layer name	output size	Layer
conv1	$64 \times 64 \times 64$	$7 \times 7 \times 7, 64$
conv2	$64 \times 64 \times 64$	$\begin{bmatrix} 1 \times 1 \times 1, 16 \\ 3 \times 3 \times 3, 16 \\ 1 \times 1 \times 1, 64 \end{bmatrix} \times 2$
conv3	$32 \times 32 \times 32$	$\begin{bmatrix} 1 \times 1 \times 1, 32 \\ 3 \times 3 \times 3, 32 \\ 1 \times 1 \times 1, 128 \end{bmatrix} \times 2$
conv4	$16 \times 16 \times 16$	$\begin{bmatrix} 1 \times 1 \times 1, 64 \\ 3 \times 3 \times 3, 64 \\ 1 \times 1 \times 1, 256 \end{bmatrix} \times 2$
conv5	$8 \times 8 \times 8$	$\begin{bmatrix} 1 \times 1 \times 1, 128 \\ 3 \times 3 \times 3, 128 \\ 1 \times 1 \times 1, 512 \end{bmatrix} \times 1$
	$1 \times 1 \times 1$	average pool, 1-d fc
Weight		1,153,441

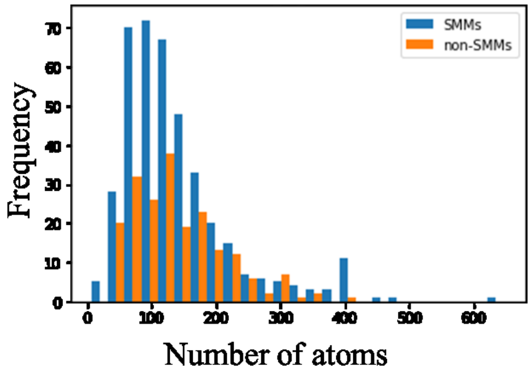

(a)

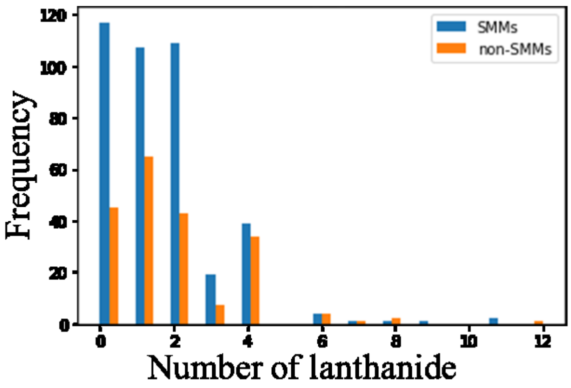

(b)

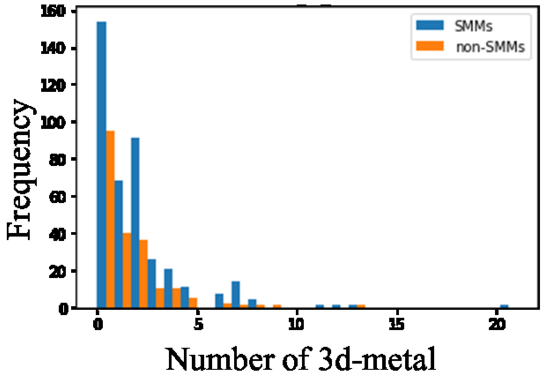

(c)

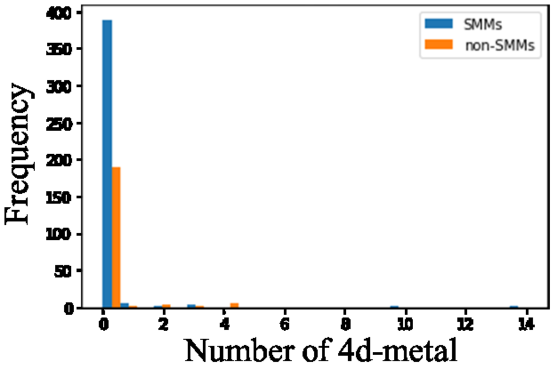

(d)

Figure S1 **Figure S1** (a) Number of atoms, (b) Number of lanthanide nuclei, (c) Number of 3d metal nuclei, (d) Number of 4d metal nuclei.

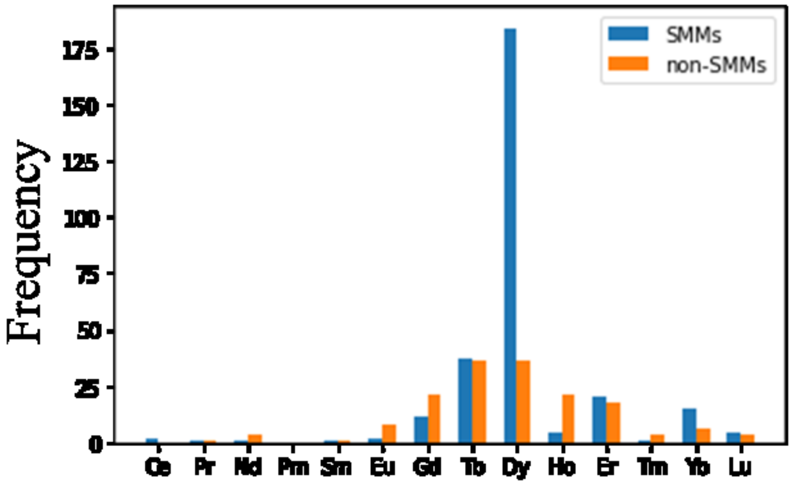

Figure S2 (a)(b) (c)(d)

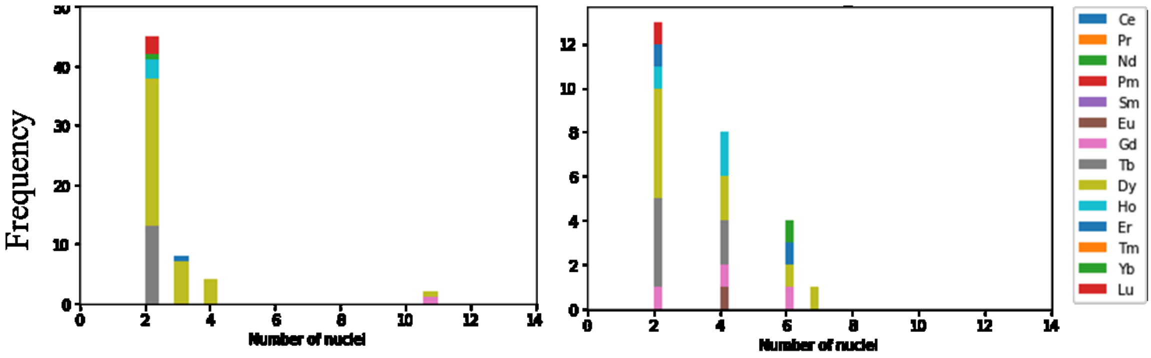

(e)

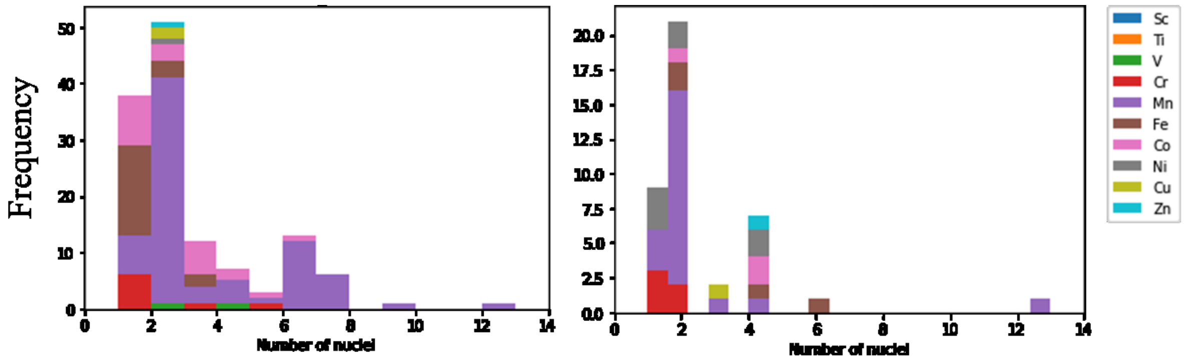

Figure S3 **Figure S2**(a) Lanthanide ions (b) Number of lanthanide nuclei in SMM molecules, (c) Number of lanthanide nuclei in non-SMM molecules, (d) Number of d metal nuclei in SMM molecules, (e) Histogram of the number of d metal nuclei in non-SMM molecules.

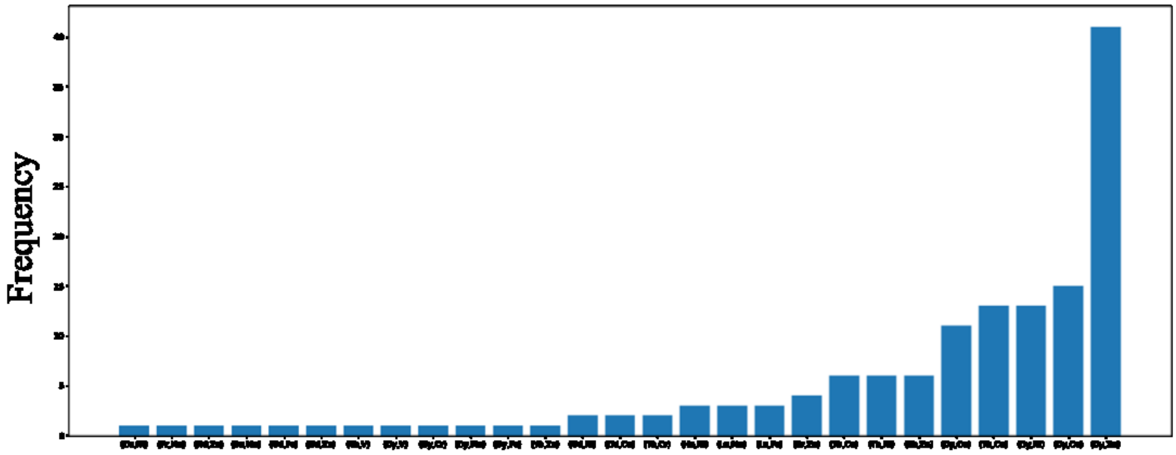

Figure S3 Each 3d-4f metal combinations SMM molecules.

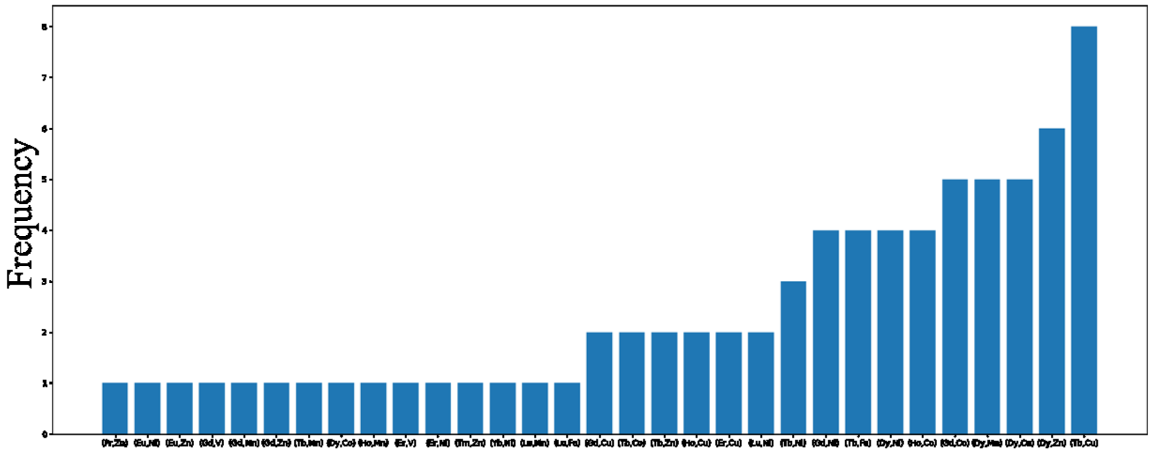

Figure S4 Figure S4 Each 3d-4f metal combinations in non-SMM molecules.

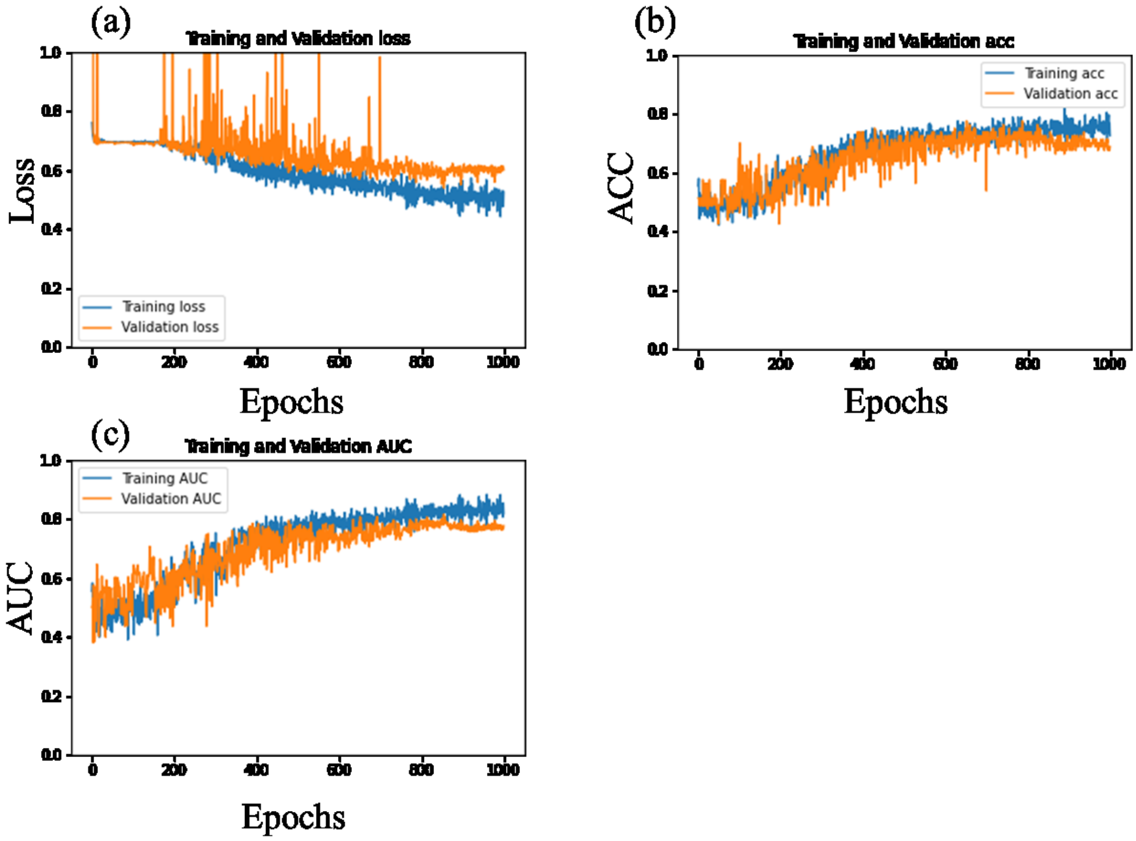

Figure S5 **Figure S5** Cross-entropy error (a) and correct answer rate (b) and AUC (c) as a function of the number of training epochs for the training dataset (blue trace) and validation dataset (orange trace).

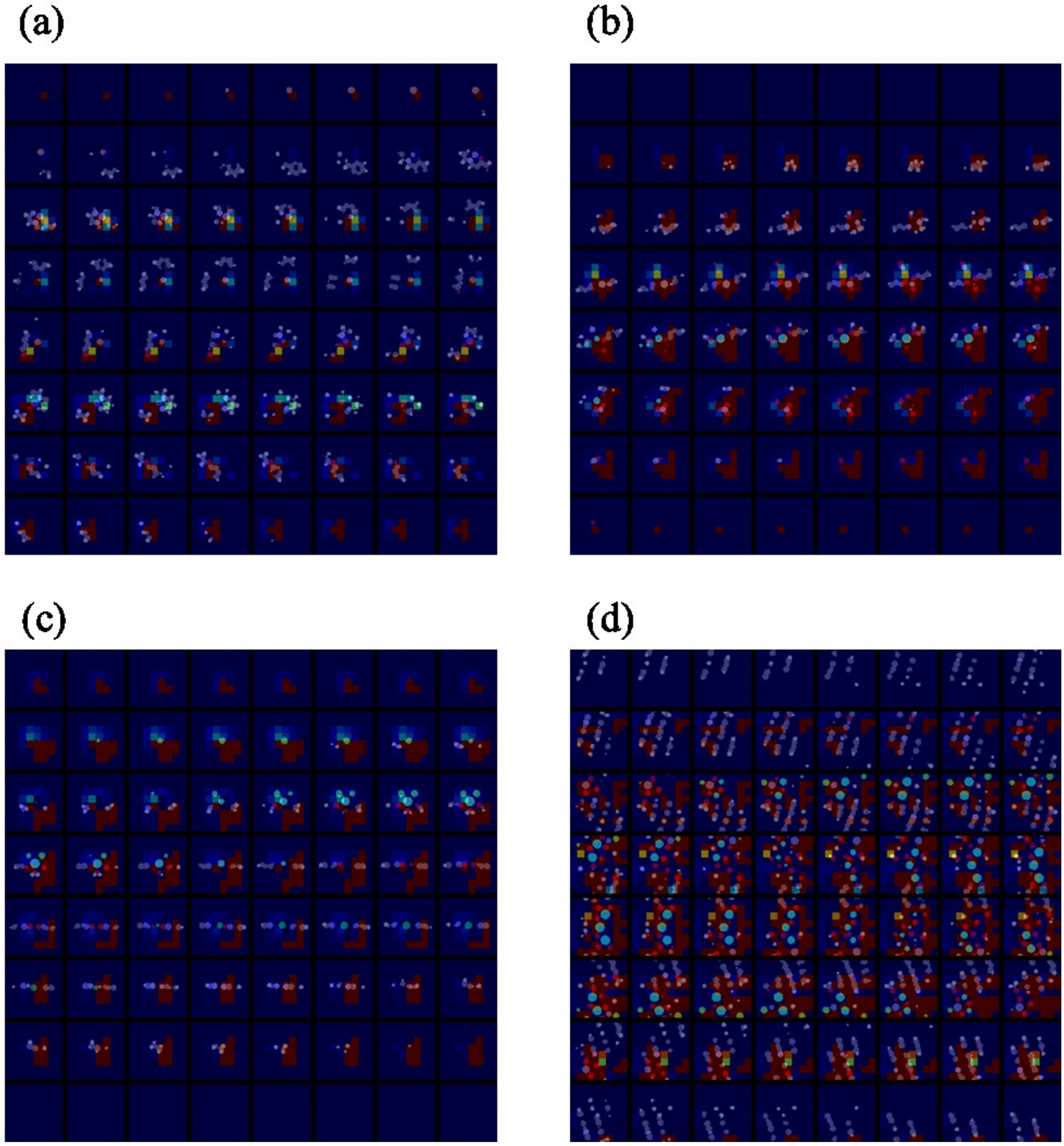

Figure S6 Visualization of CNN prediction results using Grad-Cam. a) Prediction: SMM, Result: SMM, b) Prediction: non-SMM, Result: non-SMM, c) Prediction: non-SMM, Result: SMM, d) Prediction: SMM, Result: non-SMM.
